# An observational study on intracutaneous sodium storage in intensive care patients and controls

**DOI:** 10.1371/journal.pone.0223100

**Published:** 2019-10-03

**Authors:** Marjolein van IJzendoorn, Jacob van den Born, Ryanne Hijmans, Rianne Bodde, Hanneke Buter, Wendy Dam, Peter Kingma, Gwendolyn Maes, Tsjitske van der Veen, Wierd Zijlstra, Baukje Dijkstra, Gerjan Navis, Christiaan Boerma

**Affiliations:** 1 Intensive Care, Medical Centre Leeuwarden, Friesland, the Netherlands; 2 Department of Internal Medicine–Nephrology, University Medical Centre Groningen, Groningen, the Netherlands; 3 Department of Orthopaedic Surgery, Medical Centre Leeuwarden, Friesland, the Netherlands; Yale University, UNITED STATES

## Abstract

The development of ICU-acquired sodium disturbances is not fully understood. Alterations in non-osmotic skin sodium storage, hypothetically inflammation-driven, could play a role. To investigate this in critically ill patients we conducted a patient-control study with skin punch biopsies in patients with sepsis (n = 15), after coronary artery bypass grafting (CABG, n = 15) and undergoing total hip arthroplasty (THA-controls, n = 15) respectively, together representing a range in severity of systemic inflammation. Biopsies were taken within 24 hours (sepsis) and within 2 hours (CABG) after ICU-admission, and prior to arthroplasty. Biopsies were analysed for sodium content. In addition immunostainings and quantitative real time PCR were performed. The primary aim of this study was to detect possible differences in amounts of cutaneous sodium. The secondary aims were to quantify inflammation and lymphangiogenesis with concomitant markers. The highest amounts of both water and sodium were found in patients with sepsis, with slightly lower values after CABG and the lowest amounts in THA-controls. Correlation between water and sodium was 0.5 (p<0.01). In skin biopsies in all groups comparable amounts of macrophages, T-cells and lymph vessels were found. In all groups comparable expression of inflammation markers were found. However, higher mRNA transcript expression levels of markers of lymphangiogenesis were found in patients with sepsis and after CABG. The conjoint accumulation of water and sodium points towards oedema formation. However, the correlation coefficient of 0.5 leaves room for alternative explanations, including non-osmotic sodium storage. No signs of dermal inflammation were found, but upregulation of markers of lymphangiogenesis could indicate future lymphangiogenesis.

## Introduction

Different mechanisms are involved in human sodium homeostasis. Some of these mechanisms are well-known, such as for instance fluid intake by stimulation of thirst and renal sodium and water retention and excretion. Lately, newly-found evidence has indicated that sodium storage might also play an important role in this process. Sodium storage occurs with concomitant water accumulation (osmotically active sodium storage), but it also occurs also non-osmotically. This non-osmotically active storage takes place in the so called third compartment. This compartment was already described early in the 20^th^ century [1–3].

Skin is part of the third compartment. Recently Titze et al. demonstrated and extensively described a subcutaneous compartment where sodium is non-osmotically stored [4–7]. In this compartment sodium is non-osmotically bound to glycosaminoglycans (GAGs), by pathways involving macrophages as well as lymphangiogenesis [8]. Both macrophages and lymphangiogenesis play a role in the process of inflammation [9]. Consequently, it is known that inflammation has modifying effects on GAGs [10–12]. Inflammation is common in critically ill patients, as are derangements in sodium homeostasis[13].

We hypothesize that inflammation in critically ill patients alters cutaneous sodium storage and thereby contributes to the development of sodium disorders. However, no literature about cutaneous sodium storage in critically ill patients in itself is available. Therefore we designed a study to investigate cutaneous sodium storage in critically ill patients. In the present study we measured skin sodium content and inflammatory pathways in intensive care patients with sepsis, in patients after coronary artery bypass grafting (CABG), and otherwise healthy patients undergoing primary total hip arthroplasty (THA) for arthrosis (controls). These groups were selected to represent a range of severity of inflammation, with the most severe cases being found in patients suffering from sepsis and the least severe ones in patients after CABG and absent in THA-controls.

Previous studies on intracutaneous sodium storage in humans made use of MRI to visualize and assess sodium [7,14]. This technique requires MRI with a strong magnetic field with specialized software and a specific coil, limiting its availability. In the experimental setting quantification of sodium storage in full skin appeared to be successful [5]. However, this technique requires a substantial amount of tissue, which prompted us to make use of a recently developed method which allows for the quantifying of sodium concentration in far smaller tissue samples [12,15].

The primary aim of this study was to detect differences in amounts of cutaneous sodium storage between patients with sepsis and the control groups. Said aim allowed us to divide our interest, making way for secondary aims which were as follows: to investigate the density of lymph capillaries, and presence and incidence of macrophage influx in skin biopsies of all aforementioned groups.

## Materials and methods

### Design and setting

We conducted a single center observational patient-control study in three groups of patients. The primary study population consisted of patients with sepsis. All consecutive patients with sepsis admitted to the ICU were screened for their eligibility based on the in- and exclusion criteria as presented in Table 1. Patients in this group were included within 24 hours after admission to our 20-bed mixed medical and surgical ICU. Sepsis was defined according to the sepsis-3 definition: patients had life-threatening organ dysfunction (increase in sequential organ failure assessment (SOFA) score of ≥ 2 points), caused by a dysregulated host response to infection [16]. After inclusion of a patient with sepsis two control patients (1 CABG, 1 control) were recruited, with matched gender and who fell within an age range not exceeding five years either senior or junior. These criteria were based on the previous findings that intracutaneous sodium storage is different between males and females and alters with ageing [17,18]. In all subjects two full-thickness punch skin biopsies (diameter 3mm) were performed in either the right or left hip region. In patients with sepsis biopsies were obtained within 24 hours after ICU-admission. Patients with sepsis were initially resuscitated with early goal directed therapy following the guidelines from the Surviving Sepsis Campaign. No corticosteroids were administered. In patients that underwent CABG biopsies were taken within 2 hours after completion of the surgical procedure, primarily due to the fact that during this period patients were still anesthetized. In orthopedic patients the study procedure was performed after induction of regional or general anesthesia but before start of surgery. No complications of skin punch biopsies were reported. Furthermore a spot urine sample and a blood sample were collected, with the latter one being used to measure levels of sodium, urea and creatinine. In urine sodium and creatinine were measured. Fractional excretion of sodium (FE_na_) was calculated according to the following formula:

$FEna=\frac{uNa\;x\;sCreat}{sNa\;x\;uCreat}$**Eq 1**: Fractional sodium excretion

uNa: urinary sodium concentration, sCreat: plasma creatinine concentration, sNa: serum sodium concentration, uCreat: urine creatinine concentration

**Table 1:** In- and exclusion criteria.

Inclusion	Exclusion
Sepsis group: 50–80 years	Absence of both upper legs
CABG and THA: 45–85 years	Cutaneous disease that makes biopsy of healthy skin impossible
Sepsis group: fulfilling the sepsis-3 criteria	Use of dermatocorticosteroids on the total surface of both upper legs within the previous 2 weeks
Controls: no medical history of chronic and / or systemic diseases	Fully tattooed surface of both upper legs
	Use of diuretics in the previous month
	Current dependency on renal replacement therapy

CABG: Coronary artery bypass grafting, THA: Total hip arthroplasty

Informed consent was obtained from patients or their next of kin according to applicable Dutch laws. In patients with sepsis informed consent was obtained after ICU-admission, in controls this was already done previous to either a CABG or a hip replacement. All patients were treated in accordance with the declaration of Helsinki. The study protocol was approved by the local ethics board (RTPO, Regionale Toetsingscommissie Patiëntgebonden Onderzoek, NL 56729.099.16) and registered at clinicaltrials.gov (NCT02912299). The study was funded by the Stichting Intensive Care Onderzoek Friesland.

### Data collection

Several parameters were collected from all subjects: age, gender, prescribed drugs, medical history, blood pressure before surgery or septic episode (if available), length, weight, serum and urine electrolytes and data concerning kidney function. In both patients with sepsis and patients after CABG scores on severity of illness (APACHE IV and SOFA) were calculated. Furthermore, these same groups were used to perform bioelectrical impedance analyses (BIA) in order to estimate fluid status. Due to the known inaccuracy of the registration of administration of fluids in the emergency room and in conventional hospital wards, no data about actual fluid balances since admission were available. In patients suffering from sepsis, the source of sepsis and severity of illness were registered. Skin biopsies were weighed (XS204 Analytical Balance, Mettler-Toledo International Inc, USA), after which they were packed per unit in tin cans and stored at -80°C. From the spot urine samples two 2ml cups were stored at -80°C. These cups were stored in order to allow for the possibility of performing additional analyses. Samples were destroyed after completion of all analyses.

### Bioelectrical impedance analysis

BIA is a non-invasive method to estimate body composition. Two electrodes are placed on both a hand and a foot on one side of the body. A data analyzer (BIA 101 Anniversary, Akern, Germany) produces an alternating current between these electrodes. Measured decrease in voltage and delay in flow of these current are measured as resistance (R), reactance (Xc) and phase angle (PhA) [19]. From these values both fluid and nutritional state of a patient can be deducted. R and Xc are related to a patient’s length and the determination of PhA is gender dependent.

### Sodium measurement in skin biopsies

From each patient one biopsy was thawed. The thawed tissue was cut into two comparable parts and the wet weight of both parts was measured. Both parts were dried overnight at 100⁰C, after which they were weighed again in order to measure dry weights. One part was dissolved in nitric acid and consequentially diluted, and sodium content of this solution was measured by way of flame spectrometry [12,15]. The other part was ashed to measure nitrogen via thermal conduction (Dumatherm Nitrogen/Protein analyzer, C. Gerhardt UK Ltd Northamptonshire, UK). Nitrogen content of the ashed biopsies was measured as a parameter for protein content of the tissue. This protein content was used to correct for subcutaneous fat, since the assumption was that sodium is stored in skin instead of in fat, and protein is largely absent from fat tissue [20]. Sodium content of the skin is expressed as mmol sodium per mg dry weight or per mg protein.

### Staining and qRT-PCR

The remaining biopsies were used for cryo sections using a cryostat (Leica CM1950). Those sections were 4μm for immunostainings and 5μm for quantitative real time polymerase chain reactions (qRT-PCR). Sections for immunostainings were dried for one hour and thereafter stored at -80⁰C. Samples were stained for lymphatic endothelium (Podoplanin and lymph vessels), CD^3+^ T-lymphocytes and macrophages (CD68). Details of the staining procedures are given in Table 2. Sections were assessed using a fluorescent microscope (Leica DM 400B). Photos were taken with the Leica DFX345FX camera and LAS software. Counting of lymph vessels was done blinded and manually by two researchers. T-cells and macrophages were analyzed by digital image analyses using Image J. Sections for PCR were used for RNA isolation using the Rneasy microkit (Qiagen, Venlo, The Netherlands) followed by complementary DNA (cDNA) synthesis using the Quantitect kit (Qiagen). This cDNA was used for qRT-PCR with different primer pairs. Used primers were chemokine ligand 2 (CCL2), vascular cell adhesion molecule (VCAM), vascular endothelial growth factor (VEGFC), podoplanin (PDPN) and the housekeeping gene B-actin. Details of the primers are given in Table 3. PCR was run using the FastStart Universal Sybr green (ROX) master mix (Roche, Basel, Switzerland).

**Table 2:** Details of the immunofluorescence stainings.

Cells	Pre-treatment	First antibody	Secondary and tertiary antibodies	Visualization
**T-cells**	100% acetone fixation + 0.03% H_2_O_2_ block + 1% BSA block	Rb anti-human CD3 1:200 in PBS/1%BSA (A04522 DAKO)	Goat anti-Rb IgG-HRP 1:100 in PBS/1%HS (DAKO) + Rb anti-goat IgG-HRP 1:100 in PBS/1%BSA (DAKO)	Tetramethylrhodamine-TRITC 1:50 (PerkinElmer) + DAPI 1:5000 + Cityfluor mounting medium
**Macrophages**	100% acetone fixation + 0.03% H_2_O_2_ block + 1% BSA block	Mouse anti-human CD68 1:1000 in PBS/1%BSA (EBM11 DAKO)	Rb anti-mouse IgG-HRP 1:100 in PBS/1%HS (DAKO) + Goat anti-Rb IgG-HRP 1:100 in PBS/1%HS (DAKO)	Tetramethylrhodamine-TRITC 1:50 (PerkinElmer) + DAPI 1:5000 + Cityfluor mounting medium
**Lymph endothelial cells**	100% acetone fixation + 0.03% H_2_O_2_ block + 1% BSA block	Mouse anti-human podoplanin 1:100 in PBS/1%BSA (D240 ThermoFisher)	Rb anti-mouse IgG-HRP 1:100 in PBS/1%HS (DAKO) + Goat anti-Rb IgG-HRP 1:100 in PBS/1%HS (DAKO)	Tetramethylrhodamine-TRITC 1:50 (PerkinElmer) + DAPI 1:5000 + Cityfluor mounting medium

**Table 3:** Primers used for qRT-PCR.

Process	Primer	Forward	Reverse
Inflammation	CCL2	5'-AGACTAACCCAGAAACATCC-3'	5‘-ATTGATTGCATCTGGCTG-3'
	VCAM	5'-TCCTGAGCTTCTCGTGCTCTATT-3'	5‘-TGACCCCTTCATGTTGGCTT-3'
Lymphangio-genesis	VEGFC	5'-CTGGCTCAGGAAGATTTTATG-3'	5'-TGTTTTTACAGACACACTGG-3'
	PDPN	5'-AAGATGGTTTGTCAACAGTG-3'	5'-GTACCTTCCCGACATTTTTC-3'
Housekeeping Gene	B-actine	5’- CCAACCGCGAGAAGATGA- 3’	5’- CCAGAGGCGTACAGGGATAG- 3’

### Statistical analysis

To our knowledge, skin sodium in critically ill patients was never investigated before, which consequently did not allow us to perform a power calculation. We expected small variations in the investigated variables within groups and rather more considerable variations between the three groups as separate entities. In previous animal studies, in which skin sodium was investigated, group sizes ranged from 6 to 20 animals per group [5,21–24]. Because of this study we expected that 15 patients per group would be sufficient in order to detect statistically significant differences. Statistical analyses were performed with SPSS 24 and 25 (IBM, New York, USA). Due to the small populations we used non-parametric tests (Mann-Whitney U test, Kruskal-Wallis test) to compare groups. Results are expressed as median with interquartile ranges. The correlation between sNa and cutaneous sodium concentration (cNa) was tested with chi-square and Spearman’s r coefficient (ρ) and corrected for group analysis. For all statistical analyses a p-value of < 0.05 is considered statistically significant.

## Results

### Patient characteristics

Patients were included in the three aforementioned groups between November 2016 and September 2017. Characteristics of the included patients are given in Table 4. This table shows that patients with sepsis were severely ill, according to their APACHE IV and SOFA scores. All included patients had normal sNa. According to the difference in (fractional) sodium excretion patients with sepsis did retain sodium when compared to patients after CABG and THA-controls. Patients with sepsis had markedly lower urinary sodium excretion with concomitant low FE_na_.

**Table 4:** Characteristics of included patients. Patients with sepsis included within 24 hours after ICU admission, patients after CABG included within 2 hours after ICU admission and patients with hip replacement included before start of surgery.

Variable		Sepsis (n = 15)	CABG (n = 15)	Hip replacement (n = 15)	P-value
Age, years		63 [60–75]	68 [63–74]	63 [58–72]	0.33
Male, n (%)		9 (60)	9 (60)	9 (60)	1
BMI		24 [22–26]	28 [25–30]	26 [24–30]	0.13
Source of sepsis	Abdominal	8 (53)	NA	NA	NA
	Pulmonary	3 (20)	NA	NA	NA
	Other	4 (27)	NA	NA	NA
APACHE IV, score		77 [62–90]	47 [43–54]	NA	< 0.01*
APACHE IV, %		23 [15–49]	0.8 [0.3–1.2]	NA	< 0.01*
SOFA, admission		9 [6–10]	4 [3–5]	NA	< 0.01*
SBP, mmHg		135 [128–146]	134 [123–162]	141 [131–153]	0.63
DBP, mmHg		76 [72–78]	78 [68–84]	84 [82–94]	0.01*
sNa, mmol/l*		139 [137–141]	138 [137–141]	139 [138–141]	0.52
sCreat, μmol/l*		75 [54–113]	80 [72–87]	71 [60–83]	0.46
BUN, mmol/l*		8.7 [6.5–10.8]	5 [4.3–5.8]	5.5 [3.8–6.6]	< 0.01*
uNa, mmol/l*		23 [10–76]	82 [57–96]	101 [72–151]	< 0.01*
uCreat, mmol/l*		8.7 [5.9–11.7]	2.8 [2.4–5.7]	8.9 [4.7–12.8]	< 0.01*
FE_na_, %*		1.5 [0.5–6.6]	9.6 [7.5–12.4]	6.3 [4.3–9.9]	0.01*
CRP on admission, mg/l*		132 [112–218]	NA	NA	NA
Phase angle, ⁰*	Men	4.4 [4–4.6]	5.2 [4.5–5.4]	NA	0.11
	Women	3.1 [2.9–6.4]	5.6 [4.8–6.2]	NA	0.25
Resistance, Ω/m*		238 [206–267]	268 [249–296]	NA	0.05
Reactance, Ω/m*		18 [14–21]	25 [22–30]	NA	< 0.01*
Use of ACEI/ARB, n (%)		3 (20)	4 (27)	5 (33)	0.71
Use of NSAID, n (%)	ASA excluded	0 (0)	1 (7)	6 (40)	< 0.01*
	ASA included	4 (27)	13 (87)	9 (60)	< 0.01*

Data are expressed as median [IQR], unless otherwise stated. P-value < 0.05 is considered statistically significant. Significant values are flagged with *. CABG: Coronary artery bypass grafting, BMI: Body mass index, APACHE IV: Acute physiology and chronic health evaluation–version 4 (% = predicted mortality), SOFA: Sequential organ failure assessment, SBP: Systolic blood pressure, DBP: Diastolic blood pressure, sNa: Serum sodium concentration, sCreat: Serum creatinine concentration, BUN: Blood urea nitrogen / serum urea concentration, uNa: Urine sodium concentration, uCreat: Urine creatinine concentration, ACEI: Angiotensin converting enzyme inhibitor, ARB: Angiotensin II receptor blocker, ASA: acetylsalicylic acid, NA: Not available

*Measured around time point of skin biopsies

### Skin sodium and water content and their clinical correlates

**Fig 1:**
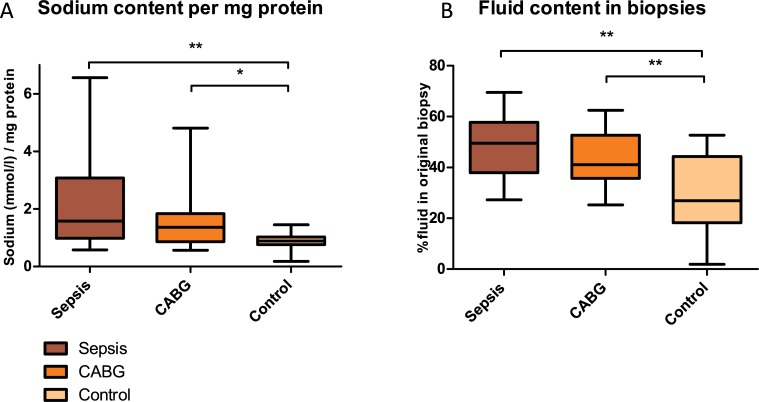
**A) Sodium and B) water content in skin biopsies.** *: P < 0.05, **: P < 0.01.

Water and sodium content were higher both in patients with sepsis and CABG patients in comparison with THA-controls (Fig 1). Water content was 50 [38–58]% in patients with sepsis, 41 [36–53]% in patients after CABG and 27 [18–44]% (p < 0.01) in THA-controls. Sodium content, expressed as mmol sodium per mg protein, was 1.6 [1–3.1], 1.4 [0.9–1.8] and 0.9 [0.8–1] (p = 0.02) in respectively septic patients, patients after CABG and THA-controls. Sodium concentrations in dry weight biopsies were 1 [0.9–1.5], 1 [0.6–1.4] and 0.7 [0.6–0.8] mmol/mg (p < 0.01) in respectively patients with sepsis, patients after CABG and THA-controls. Sodium concentration in biopsies, expressed in mmol per mg wet weight, did not differ between groups either.

**Fig 2:**
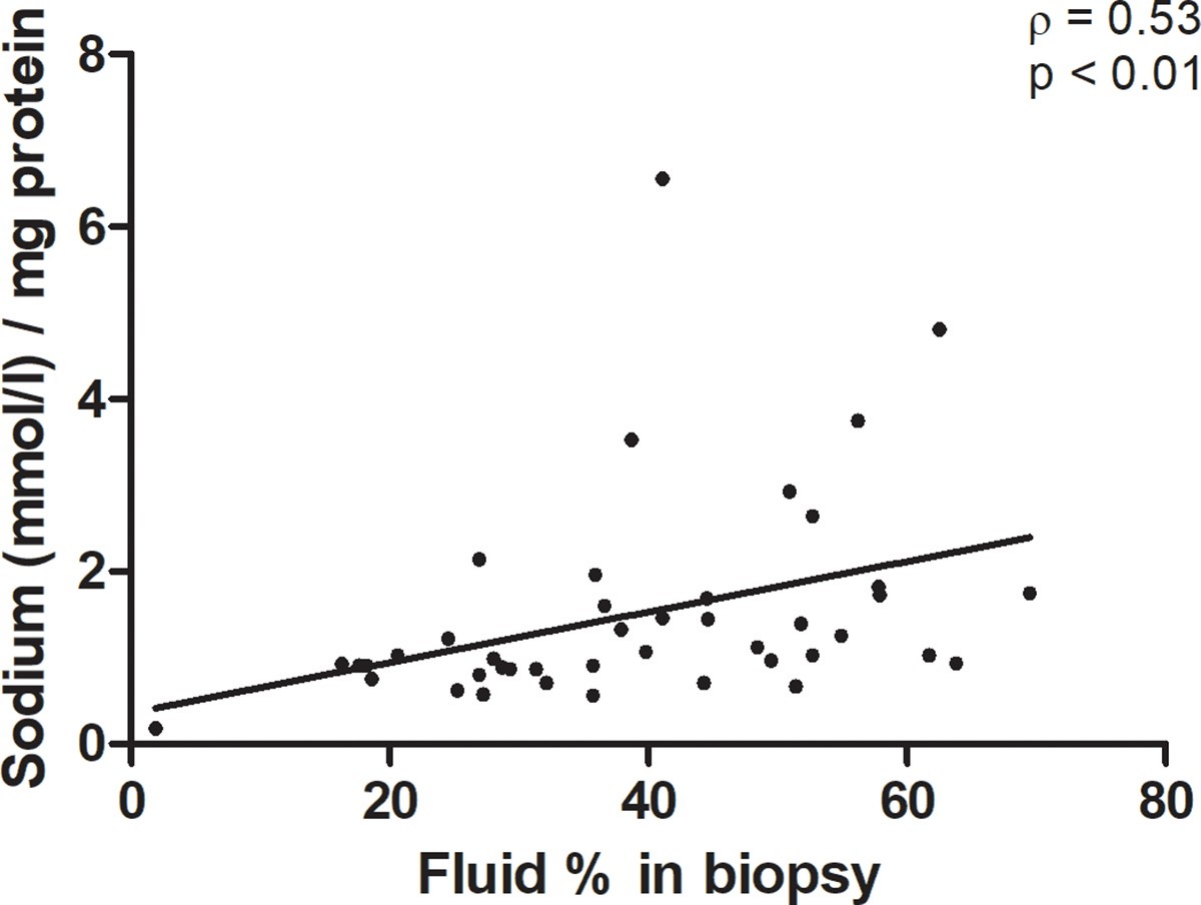
Correlation between sodium content in mmol / mg protein and fluid percentage in biopsies.

Pearson’s correlation between sodium content per mg of protein and fluid percentage in biopsies, filtered by group, was 0.5 (p < 0.01). This correlation is visualized in Fig 2. No significant correlations between sNa and cNa or uNa, or between sNa, cNa, uNa and body fluid content as measured with BIA were found. R and Xc, both corrected for length, were lower in patients with sepsis compared to CABG (p<0.01). These findings indicate higher body fluid levels. FE_na_ was 1.5 [0.5–6.6] % in patients with sepsis, 9.6 [7.5–23.4] % in patients after CABG and 6.3 [4.3–9.9] % in THA-controls (p < 0.05 between all groups). As described previously, no adequate total in hospital fluid balances were available. Total ICU-fluid intake and excretion (fluid balances) previous to the moment of inclusion were only available for patients with sepsis and patients after CABG. Patients in these groups had comparable fluid balances (patients with sepsis 1.3 [0.7–3] L positive, patients after CABG 1.5 [1.4–2] L positive, p = 0.87).

### Skin inflammatory parameters

**Fig 3:**
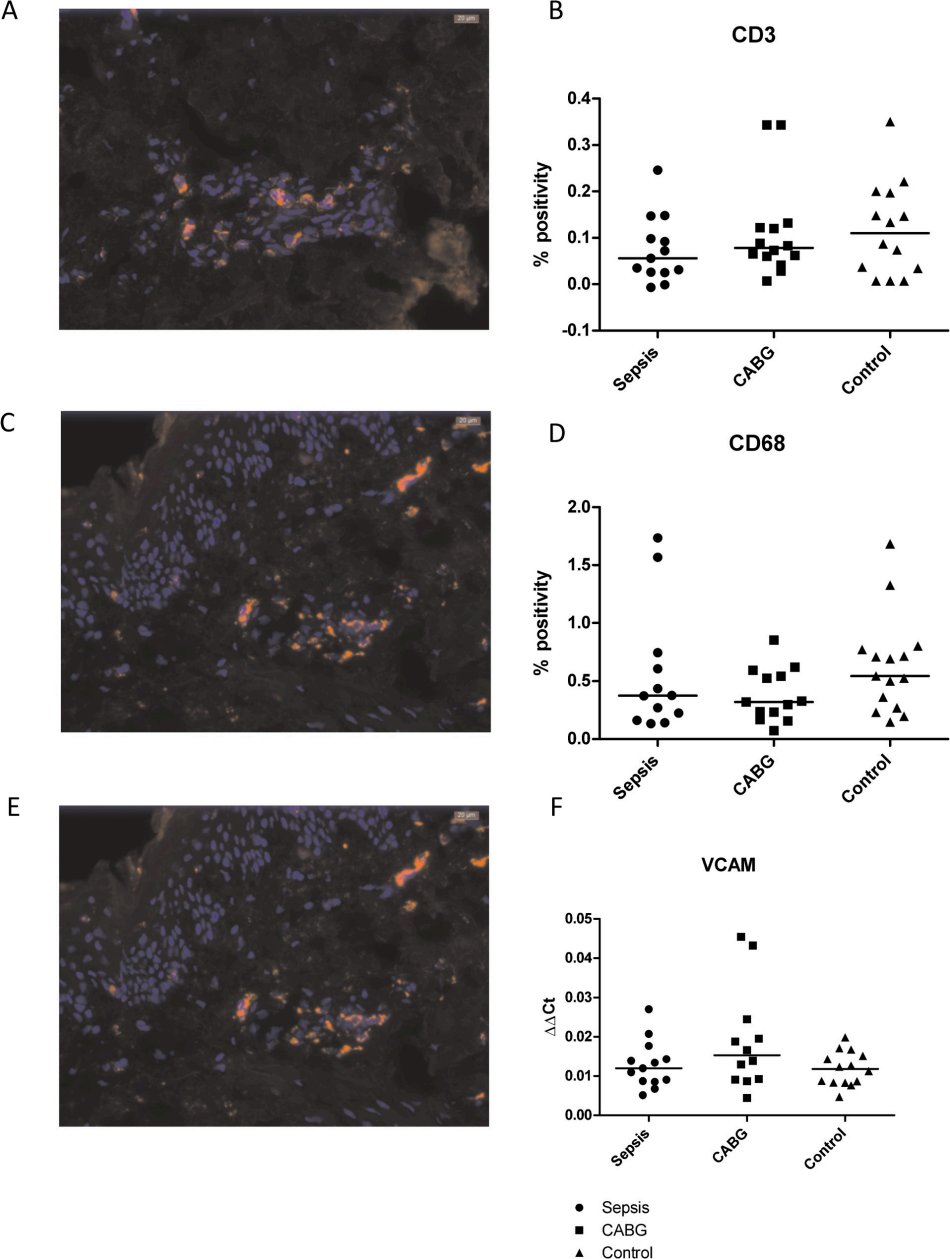
**Markers of inflammation with A) representative photo of macrophages, B) quantification of macrophages, C) representative photo of T-cells, D) quantification of T-cells, E) qRT-PCR of chemokine ligand 2 (CCL2) and F) VCAM.** In A and C, macrophages and T-cells are stained in red, nuclei (by DAPI staining) in blue.

In 4 cases (2 samples from patients with sepsis and 1 sample from both a patient after CABG and a control) not enough tissue was available for staining. In 3 additional cases not enough material was available for qRT-PCR (2 CABG, 1 control). To asses dermal inflammation we evaluated macrophages by CD68 and T-cells by CD3 staining. Both stainings showed scattered occasional macrophages and T-cells in the dermal layer of the skin biopsies. Quantification revealed no significant differences in macrophage and T-cell density among the three groups (Fig 3A–3D). We also evaluated the mRNA expression of the CCL2 (MCP1), a potent chemoattractant for monocytes/macrophages, and of VCAM-1, an endothelial adhesion molecule involved in leukocyte recruitment. qRT-PCR analyses showed no differences between patients with sepsis and THA-controls (Fig 3E and 3F). C-reactive protein (CRP), as a marker of generalized inflammation, was only measured in patients with sepsis. CRP on admission was 132 [112–218] mg/l and 236 [123–398] mg/l after 24 hours.

**Fig 4:**
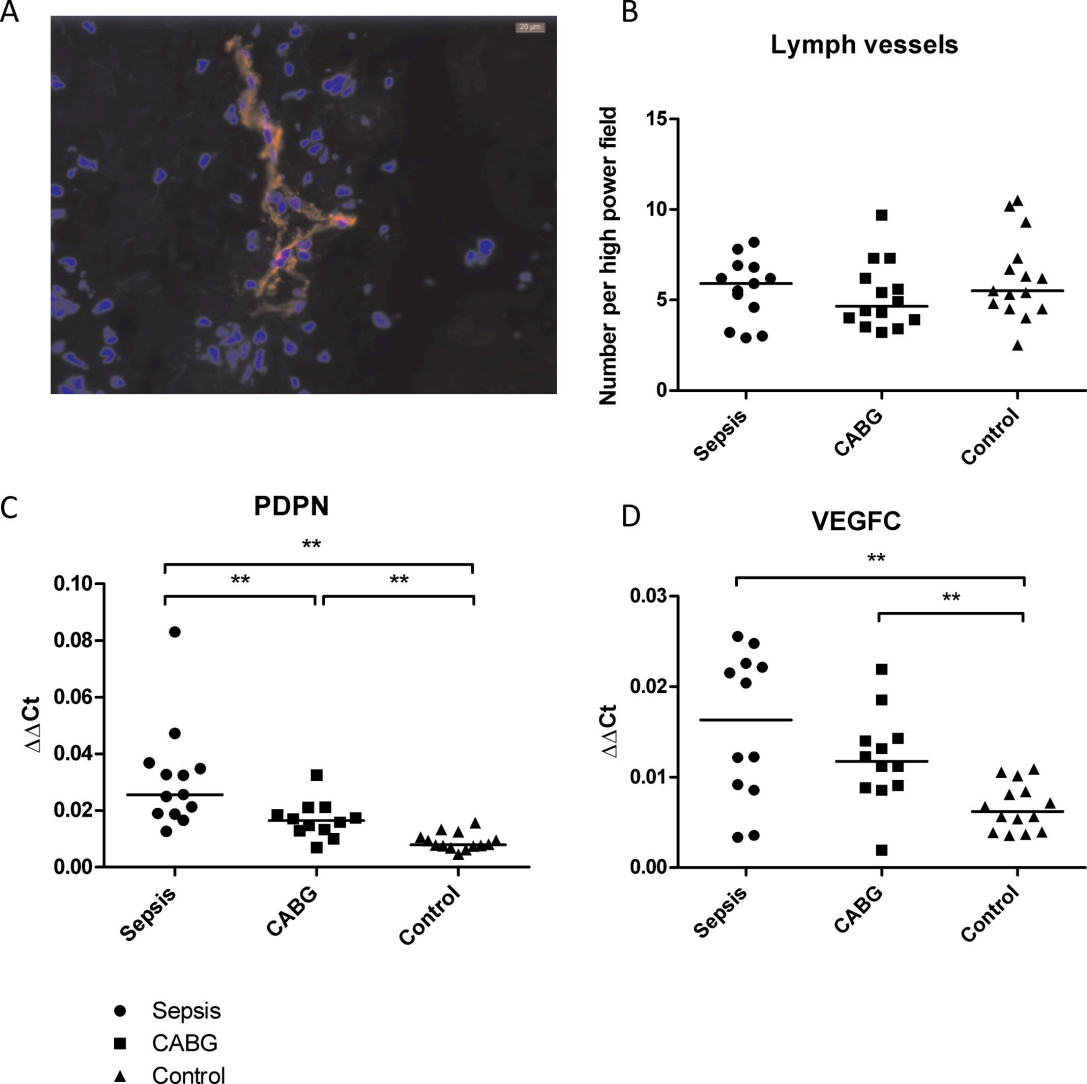
**Lymph vessels with A) representative photo of lymph vessels in red, B) it’s quantification, C) qRT-PCR for podoplanin (PDPN) and D) qRT-PCR for vascular endothelial growth factor C (VEGF-C).** *: P < 0.05, **: P < 0.01.

Furthermore we evaluated lymph vessel density by podoplanin, and lymphangiogenesis by VEGF-C, since lymph vessels have been described to be involved in dermal sodium handling. Staining of podoplanin revealed lymph vessels to be found in the dermal layer of the skin, slightly concentrated towards the epidermal/dermal junction. Manual counting of the lymph vessels in all biopsies (expressed per high power field) did not reveal differences among the three groups (Fig 4A and 4B). qRT-PCR for PDPN and VGEF-C clearly showed both transcripts to be increased in the CABG group and even more in the sepsis group, indicating lymphangiogenesis in both groups (Fig 4C and 4D). Nine patients with sepsis developed hypernatremia (defined as serum sodium concentration of ≥ 143mmol/l). No differences were found in cutaneous sodium storage, nor in markers of inflammation compared to the patients who did not develop hypernatremia.

## Discussion

Our findings suggest that a substantial proportion of cutaneous sodium content was osmotically active due to the fact that the observed differences in dermal sodium content were associated with differences in fluid content. In other words: oedema and salt accumulation go hand in hand. This hypothesis is supported by 2 findings. Firstly, the amount of sodium expressed as concentration in wet-weight samples did not differ between groups. Secondly, BIA also revealed higher body fluid levels in patients with higher sodium and fluid content in their skin. Clearly, this is in line with the current literature. Already in the first century before Christ Celcus described edema (‘tumor’) as one of the characteristics of inflammation. It is well known that both in patients with sepsis and in patients after CABG a systemic inflammatory response is present [25]. This response is reflected by the high CRP-level in patients with sepsis. Unfortunately, no CRP-levels were available for the other study groups. Inflammation in the control group is unlikely because study procedures were to be conducted before start of surgery and no THA was conducted whenever clinical signs of inflammation were present. It is of note that this inflammatory process was not reflected in the skin by macrophage or T-lymphocyte influx, nor in upregulation of inflammatory markers. In a recent study skin biopsies of healthy subjects were compared with biopsies of patients with chronic kidney disease [15]. In these chronically ill patients no changes in non-osmotic sodium storage were found. However, both inflammation and lymphangiogenesis were present. In our study levels of VCAM and CCL2, both involved in attraction of leucocytes and thereby a marker for inflammation, did not differ between patients with sepsis and controls [26,27]. The significantly lower levels of CCL2 in the CABG cohort were probably due to the routine administration of dexamethasone in these patients. On the other hand upregulation of VEGFC and PDPN, both markers of lymphangiogenesis, was found [17,28]. This upregulation may either be inflammation driven or the (concomitant) result of oedema formation by the accumulation of water and sodium respectively [18,19]. Lymphangiogenesis in inflammation is controlled by cells from the mononuclear phagocyte system (MPS) [19]. In addition, sodium itself influences lymphangiogenesis via an MPS-mediated, but inflammation-independent, pathway [19]. Data regarding sodium intake in our study population was not available but previous studies showed high sodium intake in critically ill patients [29,30]. A sign of a potential positive sodium balance in patients with sepsis in this study is a markedly lower FE_na_ in comparison to patients after CABG and THA-controls.

However, the correlation coefficient between fluid and sodium content per mg protein (ρ = 0.5, corrected for group division) suggests a moderate relationship, whilst also suggesting that not all sodium was osmotically active. If this would have been the case, a stronger correlation could be expected. Our data are not sufficient to determine why this correlation is this far below 1. (Part of) an explanation could be an interindividual difference in nature of sodium storage, which would be either osmotically or non-osmotically active.

From our data it becomes clear that the developed technique is able to quantify sodium content in small skin biopsies of various patient categories. In each patient category sodium concentrations were within narrow limits, despite the absolute low values. In addition, there was a clear separation between groups.

The main limitation of this study is that we did not detect changes in non-osmotic sodium storage with this particular method. Another limitation turned out to be the fact that skin biopsies in patients with sepsis were taken in the early phase of severe disease. The inflammation found in patients with chronic kidney disease suggests that, on a longer term, significant changes in skin biopsies of critically ill patients might be found. The markedly lower FE_na_ in patients with sepsis, and the development of hypernatremia in a substantial part of these patients, suggest sodium accumulation. Perhaps, during the course of a septic period, changes in cutaneous sodium storage might become visible as well.

## Conclusions

Both sepsis and CABG patients had significantly higher levels of fluid and sodium content in comparison to THA-controls. The modest association between sodium and water accumulation leaves space for alternative explanations instead of simply oedema formation. Besides, systemic inflammation was not reflected in skin, at least not in the early phase of sepsis. With the current methodology we were able to detect differences in sodium storage in small sample skin biopsies, applicable in the clinical setting. These data allows for further research on sodium handling in critically ill patients, for example with consecutive skin biopsies during the course of critical illness.
